# AMaLa: Analysis of Directed Evolution Experiments via Annealed Mutational Approximated Landscape

**DOI:** 10.3390/ijms222010908

**Published:** 2021-10-09

**Authors:** Luca Sesta, Guido Uguzzoni, Jorge Fernandez-de-Cossio-Diaz, Andrea Pagnani

**Affiliations:** 1 Politecnico di Torino, Corso Duca degli Abruzzi 24, I-10129 Torino, Italy; lucasesta95@gmail.com (L.S.); guido.uguzzoni@gmail.com (G.U.); andrea.pagnani@polito.it (A.P.); 2 Laboratory of Physics of the Ecole Normale Supérieure, CNRS UMR 8023 & PSL Research, Sorbonne Université, 24 rue Lhomond, 75005 Paris, France; 3 Center of Molecular Immunology, Systems Biology Department, Playa, Havana CP 11600, Cuba; 4 Italian Institute for Genomic Medicine, IRCCS Candiolo, SP-142, I-10060 Candiolo, Italy; 5 INFN, Sezione di Torino, I-10125 Torino, Italy

**Keywords:** computational biology, statistical modeling, fitness landscape, Directed Evolution, Deep Mutational Scanning, direct-coupling analysis

## Abstract

We present Annealed Mutational approximated Landscape (AMaLa), a new method to infer fitness landscapes from Directed Evolution experiments sequencing data. Such experiments typically start from a single wild-type sequence, which undergoes Darwinian in vitro evolution via multiple rounds of mutation and selection for a target phenotype. In the last years, Directed Evolution is emerging as a powerful instrument to probe fitness landscapes under controlled experimental conditions and as a relevant testing ground to develop accurate statistical models and inference algorithms (thanks to high-throughput screening and sequencing). Fitness landscape modeling either uses the enrichment of variants abundances as input, thus requiring the observation of the same variants at different rounds or assuming the last sequenced round as being sampled from an equilibrium distribution. AMaLa aims at effectively leveraging the information encoded in the whole time evolution. To do so, while assuming statistical sampling independence between sequenced rounds, the possible trajectories in sequence space are gauged with a time-dependent statistical weight consisting of two contributions: (i) an energy term accounting for the selection process and (ii) a generalized Jukes–Cantor model for the purely mutational step. This simple scheme enables accurately describing the Directed Evolution dynamics and inferring a fitness landscape that correctly reproduces the measures of the phenotype under selection (e.g., antibiotic drug resistance), notably outperforming widely used inference strategies. In addition, we assess the reliability of AMaLa by showing how the inferred statistical model could be used to predict relevant structural properties of the wild-type sequence.

## 1. Introduction

Over the last few years, the development of increasingly accurate high-throughput biochemical assays with massive parallel sequencing techniques has established large-scale genetic screening as a fundamental tool for the investigation of the relationship between evolution, fitness and other important biological concepts that were behind the experimental research [1,2,3,4,5,6,7,8,9,10,11,12,13,14,15,16,17,18,19,20,21,22,23,24,25,26,27].

These experiments that simultaneously screen up to millions of variants of a given protein come in two main flavors. Deep Mutational Scanning (DMS) experiments [8], where all the combinatorial complexity of the experiment is encoded in the initial library that undergoes several iterative steps of target selection and amplification. In contrast, in Directed Evolution experiments [28], at each round of the procedure, mutations are randomly created with a tuned mutation rate by error-prone PCR (ep-PCR). In either case, the typical pipeline sees a library of protein variants undergoing cycles of selection for functional activity. At some of the intermediate steps, a sample of the mutant population is sequenced to assess the relative variant abundances.

Recently, high-throughput screening experiments have been used to predict the folded three-dimensional structure [26,29]. It has been argued that screening experiments without mutations, such as DMS, are not able to probe the sequence space deeply enough to generate a statistically relevant signal of the folded structure. In both studies [26,29], the author’s experimental solution involves the Directed Evolution framework introducing an error-prone PCR to explore a broader sequence space region. Then, their idea is to apply Direct Coupling Analysis (DCA) to the artificially generated protein variants by the Directed Evolution experiment. DCA was originally developed to model co-evolution in homologous protein families [30,31]. It uses the inferred epistatic interaction between pairs of residues to reconstruct the contact map. In this strategy, a maximum entropy model is learned from the outcome of the last round of selection. This set of proteins is the output of a functional selection constrained to the structural properties of the wild-type, mimicking the protein families generated by natural evolution. Some of the differences between the two processes involve the time duration, the broader sequence diversity, the population sizes, the mutation rates, the variability in the cellular environments where the protein operates (e.g., different temperatures), etc.

Besides the motivation to predict the protein structure, we argue that Directed Evolution experimental data can be employed to infer a global genotype-to-phenotype mapping, or in other words, a comprehensive fitness landscape. Indeed, such experiments allow us to extensively explore the sequence space around the wild-type sequence, thanks to the continuous generation of new variants. At the same time, these variants are bound to be functional since they are subjected to a selection screening. Another typical goal of DE experiments is in fact the production of one or more specific variants that are optimal with respect to the selection process. We point out how this is conceptually different from measuring the effect of single-point mutations on fitness. In this case, no statistical model is learned, and instead, an accurate fitness score is associated to a limited pool of sequences (as in [4,7]). Nonetheless, these kind of experiments represent an important testing-ground for statistically inferred models [32], especially because it might not be easy to derive such accurate estimates of fitness/functionality in the case of DE evolution experiments. Furthermore, we remark how having a correct model of the genotype–phenotype association permits answering fundamental questions about the relationship between the fitness landscape and molecular evolution [33,34,35]; on a more practical side, it also allows us to design novel effective proteins [36,37].

As more high-throughput sequencing data of screened libraries are available, new computational methods for accurate statistical modeling of the genotype–phenotype association are actively developed. Most of the computational strategies developed so far rely on two approaches: (i) DCA-inspired models of phylogenetically related sequences used to describe local fitness landscapes [38,39,40,41,42,43,44,45] and, very recently, in [46], (ii) a supervised machine learning approach on a sequenced sample of high-throughput functional assays or screening experiments. In this case, a statistical model of the mutants’ fitness is inferred from a subset of the sequencing data (training set) with machine learning techniques developed to solve a specific—generally non-linear—regression problem [26,27,42,47,48,49,50,51].

More recently, alternative unsupervised strategies have been proposed [52,53] to cope with all sequencing information coming from screening experiments. In [53], a probabilistic model is described, which takes into account three different steps always occurring in screening experiments: (i) selection, (ii) amplification, and (iii) sequencing. Although such models are very effective for describing the Deep Mutational Scanning experiments in the absence of the mutagenesis step, it relies on the variations of the variants’ relative abundances across rounds and hence of the sample of the same variants at different time-steps with sufficient statistics. However, several screening setups and, in particular, Directed Evolution experiments do not allow for this computation. It can be due to a small sequencing depth (related to the initial library size) or to an additional error-prone PCR step to create more variability as in the Directed Evolution experiment. In the last case, new sequences appear at each round of the experimental pipeline; thus, the variation of the library composition is not only due to fitness selection but also to a stochastic drift.

In this paper, we propose Annealed Mutational approximated Landscape analysis (AMaLa), a new unsupervised inference framework that effectively takes into account the mutational aspects of Directed Evolution experiments in terms of a global likelihood to observe a time series of variants’ abundances. AMaLa specifically aims to model the whole in vitro evolutionary trajectory in contrast to the DCA approach that only considers the last sequenced round. The ultimate purpose of the method is to infer a global genotype-to-phenotype mapping, defined in terms of a multivariate Potts-like Hamiltonian with both additive contributions from individual residues and pairwise epistatic interactions. Such a mapping assigns a score that is a proxy for functionality to all possible protein sequences. We remark that the method does not require computing an enrichment ratio of the variants population.

We use the AMaLa inference scheme to analyze three Directed Evolution high-throughput experiments [26,29]. Once the global genotype–phenotype mapping (mutational landscape) is inferred, it is possible to assess its predictive power in two different ways: (i) when independent accurate fitness measurements are available one can compute their correlation with the inferred model statistical energy. Here, we relied on the measurements reported in [4,7], in which the fitness scores of the single point mutants of the TEM-1 wild-type are measured in terms of minimum inhibitory antibiotic concentration. (ii) Alternatively one can predict residue–residue contact maps, which was the original goal of [26,29]. In either case, our results are better (or equivalent when the statistical signal is too poor) than other DCA-inspired strategies, suggesting that modeling the whole evolutionary trajectory of Directed Evolution experiments allows for a more robust description of the fitness landscape. Moreover, one of the most interesting outcomes of our analysis is the prediction of how experimental strategies could be optimally tuned to produce more informative data. In particular, by running extensive simulations of in silico experiments, we show how the trade-off between mutation and selective pressure is a critical parameter that needs to be fine-tuned. In agreement with what was observed in [46], our analysis suggests that lower selective pressure in both [26,29] would have been beneficial to explore more efficaciously the fitness landscape.

## 2. New Approach

*Annealed Mutational approximated Landscape* (AMaLa) uses the sequencing samples of rounds of Directed Evolution experiments to learn a map between the protein amino acid sequence and the fitness associated with the selection process, generically indicated as the fitness landscape. Typically, fitness in these experiments is related to the binding affinity to a certain target or to more complex phenotypic traits, such as antibiotic resistance in bacterial strains.

We consider the probability of observing a generic sequence at a certain time (or round) *t* in the following form:

(1) $$P^{\left(t\right)}\left(S\right)=\frac{e^{-H^{\left(t\right)}\left(S\right)}}{Z^{\left(t\right)}},$$

where $H^{\left(t\right)}\left(S\right)$ is a time-dependent Hamiltonian function, and $Z^{\left(t\right)}=\sum_{\left\{S\right\}}\exp\left[-H^{\left(t\right)}\left(S\right)\right]$ is the associated partition function. The approximation assumes that the model probabilities of observing a given sequence at different times encoded in Equation (1) are statistically independent. An alternative approach would consider the dynamics as a Markov process, describing the probability of whole trajectories in sequence space. However, such a strategy seems to be computationally intractable, as one should sum over all possible trajectories connecting two sequences at subsequent times. Here, we consider a factorized time-dependent likelihood, which effectively takes into account the Directed Evolution dynamics as:

(2) $$L\left[\theta_{E}\right]=\sum_{t=\{t_{1},t_{2},\cdots ,T\}}\sum_{a=1}^{M^{\left(t\right)}}w^{\left(a,t\right)}\log P^{\left(t\right)}\left(S^{\left(a,t\right)}\right),$$

where $w^{\left(a,t\right)}$ is the normalized abundance of sequence $a=1,\cdots ,M^{\left(t\right)}$ at time *t*, $w^{\left(a,t\right)}=N^{\left(a,t\right)}/\sum_{a^{\prime }=1}^{M^{\left(t\right)}}N^{\left(a^{\prime },t\right)}$, and $N^{\left(a,t\right)}$ is the absolute abundance of sequence *a* at round *t*. We will implement a maximum likelihood estimate of $\theta_{E}$, which estimates the model parameters maximizing Equation (2), the likelihood of the model parameters given the data. The second important assumption is that the time-dependent Hamiltonian function depends on two terms that account for the two different processes occurring in Directed Evolution experiments: selection and mutation.

To describe the selection term, we introduce a statistical energy E(S) function of the sequence, labeled by $S=\left(\sigma_{1},\sigma_{2},\ldots ,\sigma_{L}\right)$, where *L* is the number of sites in the sequence, and $\sigma_{i}=\left\{A,C,\ldots ,Y\right\}$ is the amino acid at residue *i*. We hypothesize that the statistical energy (or more precisely, its opposite) is related to the fitness of the sequence in a selection process. In analogy with standard DCA analysis, we choose to parameterize the energy function as a generalized Potts model:

(3) $$E\left(S\right)=-\sum_{i=1}^{L}h_{i}^{\left(E\right)}\left(\sigma_{i}\right)-\sum_{i=1}^{L-1}\sum_{j=i+1}^{L}J_{ij}^{\left(E\right)}\left(\sigma_{i},\sigma_{j}\right)\;.$$

The set of parameters $\theta_{E}:=\left\{h^{\left(E\right)},J^{\left(E\right)}\right\}$ is related to the single-site residue frequency and pairs epistatic interactions [54]. The amino acids are mapped onto natural numbers $\sigma_{i}\in \left\{1,\cdots ,q\right\}$, where q=20, and i∈1,⋯,L identifies the sites along the sequence.

Concerning the random mutation process, we used a simple generalization of the Jukes–Cantor model [55] to account for amino acid substitutions instead of DNA base pairs. We introduce this approximation of the real mutation process that does not consider codon bias for simplicity, although the same strategy could be utilized in a more general context, such as, for instance, considering a probability transition matrix between codons [46]. Jukes–Cantor is a Markov model that assumes equally probable mutations among amino acids, and it is solely defined by a mutation rate μ through the following transition matrix:

(4) $$W_{l\to k}^{\left(t\right)}\left(\mu \right)=\left\{\begin{array}{cc} \frac{1-e^{-\mu t}}{q}\;\; & l\neq k,\\ \frac{1+\left(q-1\right)e^{-\mu t}}{q}\;\; & l=k, \end{array}\right.$$

where {l,k}∈1,…,q are two generic amino acids. Equation (4) defines the transition probability from amino acid *l* to *k* over a time interval *t*. In Directed Evolution experiments, the starting point is typically a single sequence, the *wild-ype*, that undergoes several rounds of error-prone PCR to create the initial library to be screened. Under the hypothesis that mutation is a site-independent process, we can express the probability of observing a sequence S at time *t* through Equation (4) as a function of $h_{D}\left(S,S^{\left(\mathrm{wt}\right)}\right):=\sum_{i}^{L}\delta \left(S_{i};S_{i}^{\left(\mathrm{wt}\right)}\right)$, i.e., its Hamming distance from the wild-type, through:

(5) $$Q^{\left(t\right)}\left(S\;|\;h_{D}\left(S,S^{\left(\mathrm{wt}\right)}\right)=d\right)=\frac{1}{Z^{\left(t\right)}}e^{-\nu (t)d},$$

where we introduced the time-dependent ν parameter:

(6) $$\nu \left(t\right)=\ln\left[\frac{1+\left(q-1\right)e^{-\mu t}}{1-e^{-\mu t}}\right].$$

The normalization factor is $Z^{\left(t\right)}=e^{-\nu (t)L}{\left[\left(q-1\right)+e^{\nu (t)}\right]}^{L}$. The asymptotic properties of the parameters ν are the following: As t→0ν diverges, in such a way that at t=0, all sequences but the wild-type have zero probability. On the other hand, ν goes to zero as t→+∞, so the asymptotic distribution is uniform in the sequence space. Note that the presented model is defined for simplicity over a continuous-time domain, while the real rounds occur at discrete times. Validation on simulated data is analyzed in Section 3.2, whereas further results on the purely mutational process (in absence of selection) are reported in the Supplementary Materials Section S2.

Combining the Jukes–Cantor mutational model with the selection process term, we obtain the Hamiltonian expression and thus the probability of finding a sequence S at time *t* as:

(7) $$P^{\left(t\right)}\left(S\right)=\frac{e^{-\beta \left(t\right)E\left(S\right)-\nu \left(t\right)h_{D}\left(S,S^{\left(\mathrm{wt}\right)}\right)}}{Z^{\left(t\right)}}.$$

Comparing Equation (7) to the case with only purely random mutation, the presence of the selection biases the variant statistics towards the fittest. As a consequence, in this regime, the Jukes–Cantor model becomes, at most, a convenient approximation. To alleviate these effects, we optimize the parameter ν(t) in Equation (6) to obtain an effective value that maximizes the pseudo-likelihood. When using Equation (7), one assumes that in the course of the Directed Evolution experiment, the consensus sequence does not drift too far away from the wild-type initial sequence. Interestingly, it turns out that in the concrete Directed Evolution experiments considered here, this assumption is approximately true.

The β(t) factor in the selection term encodes its time dependency. From the standpoint of statistical mechanics, it can be interpreted as a fictitious inverse temperature that increases with time, which inspires the term “annealing” in our method name. Let us consider an experiment in the absence of mutation steps (e.g., in the Deep Mutational Scan experiments) but in the presence of several selection rounds. We define $P_{S}\left(S\right)$ as the probability that a sequence S is selected. The probability $P^{\left(t\right)}\left(S\right)$ of observing a sequence S at round *t* is proportional to $P_{S}{\left(S\right)}^{t}$. Indeed, sequence S must survive *t* rounds of selection to be observed at round *t*. In this simple case, the inverse temperature β(t) exactly coincides with *t*. Temperature decreases with subsequent rounds, and in the theoretical limit of an infinite number of selection rounds, the only surviving sequence is the ground state of the Hamiltonian *E*, i.e., the sequence with the larger probability according to the model.

The likelihood of the whole experiment outcome is obtained by substituting Equation (7) into Equation (2). As typical in this type of inference problems, the exact maximization of the likelihood requires the determination of the partition function of the model, whose computational complexity scales as $O(q^{L})$. To overcome this limitation, instead of using the likelihood, we maximize a different but related quantity called the pseudo-likelihood. This approximation allows for a computationally efficient way to learn the parameters [56,57]. See the Supplementary Materials Section S1 for the complete definition of the pseudo-likelihood function and the regularization term.

## 3. Results

### 3.1. Directed Evolution Experiments

We tested AMaLa on three recently published Directed Evolution experiments: two are described in [29] and one in [26]. The proteins mutated and selected in these experiments belong to the β-lactamase family (PSE-1 and TEM-1) and acetyltransferase family (AAC6). The β-lactamase is responsible for the hydrolysis of antibiotics, such as penicillin, ampicillin and carbenicillin, while the acetyltransferase is responsible for the catalysis of kanamycin via acetylation. The experiment alternates rounds of variants selection and mutagenesis steps where part of the population is randomly mutated through error-prone PCR. The fitness selection is obtained by exposing bacterial cultures containing the plasmids library to a certain concentration of ampicillin in the case of PSE-1 and TEM-1 (fixed for the former and variable for the latter) and kanamycin for AAC6. In all three experiments, only a subset of the rounds after the selection step are sequenced (see Section 4.1 for more details on the experimental pipeline).

We used two strategies to test the inferred fitness landscape: (i) by direct comparison of the predicted fitness with experimental measures of the phenotype under selection in the Directed Evolution experiment for a set of variants; (ii) through indirect assessment of the predicted 3D structure of the protein using the inferred epistatic interaction of the learned model (DCA analysis [54]). The first strategy can only be applied to TEM-1 since, to the best of our knowledge, there are no published high-throughput measures of kanamycin and ampicillin resistance for the other two proteins (AAC6, PSE-1). Moreover, being able to use Directed Evolution experiments to predict the structure of a protein is clearly an interesting research perspective in itself and the main goal of both [26,29].

#### 3.1.1. Prediction of Mutation Effect on Fitness

High-throughput measurements ampicillin resistance (viz. the same phenotypic trait under selective pressure in [26]) of single-site mutants of TEM-1 are presented in [7], whereas measurements of the minimum inhibitory concentration to β-lactamase amoxicillin are presented in [4]. The fitness of the different variants is estimated as the minimum inhibitory concentration to ampicillin of the mutants with respect to the wild-type. It has to be noted that the wild-type sequence in the experiment of [26] (PDB entry 1ZG4) and the one in [7] (Uniprot-P62593) have two mismatches.

**Figure 1:**
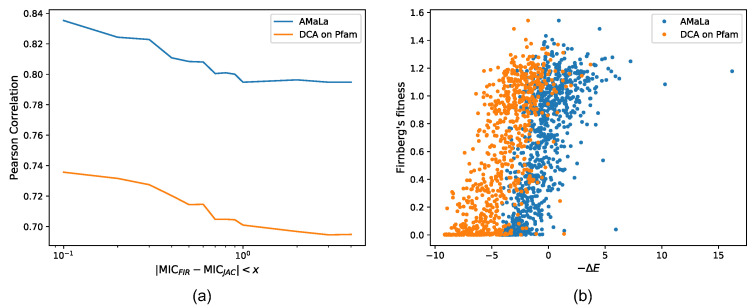
Correlation between inferred energies and fitness measurements realized in [7]. Such measurements are preliminarily mapped over [4] data following the same procedure proposed in [32]. As a consequence of this mapping, the overlap of the sequences appearing in both [26] and the testing dataset amounts to just two. Furthermore, it is also possible to filter out the noisiest data, retaining only those measurements displaying a low discrepancy between the two datasets. Panel (**a**) shows the trend of the Pearson correlation obtained as a function of this discrepancy threshold. Namely, correlations are referred to as energies inferred over Fantini’s dataset [26] via AMaLa (blue line) and over PFAM PF13354 via PlmDCA (orange). More specifically, such energies are previously mapped over fitness scores via the same procedure exploited to map [7] into [4]. This strategy allows expressing the correlation performance in terms of a linear estimator rather than the more general Spearman coefficient. From the plot, it emerges how correlations increase by progressively excluding those measurements with the highest discrepancy among the datasets. Moreover, [26] measurements analyzed via AMaLa turn out to provide a better fitness estimator with respect to the homology family, characterized by a much more dispersed distribution of sequences. In panel (**b**), the scatter between the minus energies (not mapped) and the fitness measurements of [7] is reported, with a discrepancy threshold between minimum inhibitory concentrations equal to x=1.0.

The statistical energy score inferred by AMaLa on the dataset of Fantini et al. highly correlates with the Firnberg et al. fitness measurements, with a Pearson correlation coefficient larger than ρ=0.8, suggesting that the method is able to learn a reliable fitness landscape. It is interesting to compare it with the approach outlined in [32], where a Boltzmann learning DCA-based approach is applied to the PFAM β-lactamase family (PF13354). In this case, the correlation of the experimental minimum inhibitory concentration with the statistical energy score shows a Pearson correlation coefficient of ρ∼0.7, as shown in Figure 1.

**Figure 2:**
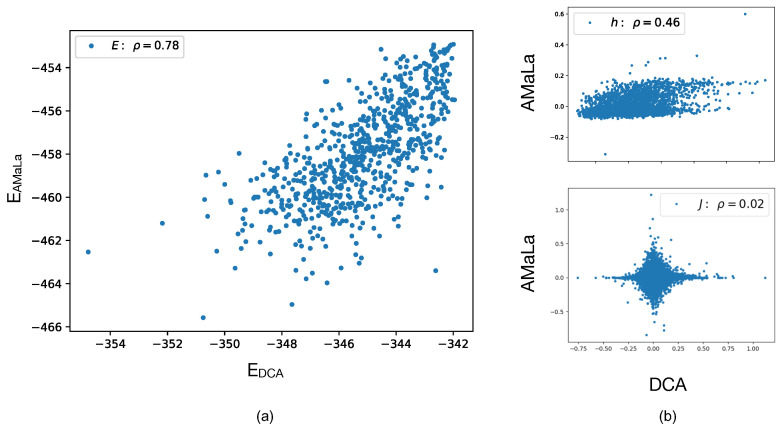
Model comparison between standard PlmDCA performed over the homology family (PF13354) and AMaLa inferred over [26] data restricted to residues corresponding to the homologues alignment. Panel (**a**) shows the scatter between the resulting total energies. Panel (**b**) displays the scatter plots of individual parameters. In the upper plot, the scatter among single-site fields h is reported, and in the lower one, among pair interaction coupling J. Even if energetic parameters display separately either low ($\rho_{h}=0.46$) or no correlation at all ($\rho_{J}=0.01$), the resulting energies are nonetheless significantly correlated, the Pearson coefficient being $\rho_{E}=0.77$. This underlines how the quantity encoding the relevant phenotypic information is indeed the total energy.

Directed Evolution experiments from Fantini et al. and the multiple sequence alignment homologous sequences contained in PF13354 provide us with two very different datasets: the first one is a *local* exploration around the wild-type, with sequences selected to the medium-low level of ampicillin selective pressure (average sequence identity of 85%), whereas the second, not surprisingly considering the extremely long time-scale involved in the evolutionary process, shows a remarkably high degree of variability (average sequence identity of 19%). Both can be used to learn a statistical model (AMaLa for [26], PlmDCA [57] for PF13354) providing two distinct sets of model parameters that, remarkably, correlate with each other in terms of the statistical energy score (see panel (a) of Figure 2) and to the fitness measurements. Interestingly, the parameters of the two models do not correlate with each other (see panels (b) in Figure 2), and consequently, they provide very different contact predictions when used to infer structure information, as outlined in the next section. We do not have a clear interpretation of this intriguing result.

#### 3.1.2. Residue Contacts Predictions

Direct coupling analysis is a powerful tool to extract structural properties from multiple sequence alignments of evolutionary-related protein sequences. However, to show its full potential, multiple sequence alignments of at least $10^{3}$ sequences must be used. For many protein families, the number of homologous sequences available from public databases (e.g., PFAM or UNIPROT) is not sufficient to obtain a reliable folding structure using DCA predictions. Thus, the question of whether one can use artificially created sequences from Directed Evolution experiments to extract structural information has a very interesting practical purpose, as discussed in [26,29]. In both papers, the authors apply two similar pseudo-likelihood-based inference strategies (the PlmDCA algorithm in [26] and EV-coupling algorithms in [29]) to learn a Potts model from the sequences in the last sequenced round of the experiment. Only one of the two experimental works [29] reached a precision sufficient to correctly fold the protein.

Here, we propose a different approach that leverages the sequencing information from all rounds of the Directed Evolution experiment. AMaLa, instead of focusing only on the final step of the in vitro Darwinian dynamics of Directed Evolution experiments, indeed utilizes the whole time series. We hypothesize that being able to analyze all available data (as opposite to the use of just the last sequenced step) through a model that explicitly (albeit in an effective way) takes into account both mutation and selection steps could, in principle, generate a more accurate model of the selection process, providing at the same time better structural information.

**Figure 3:**
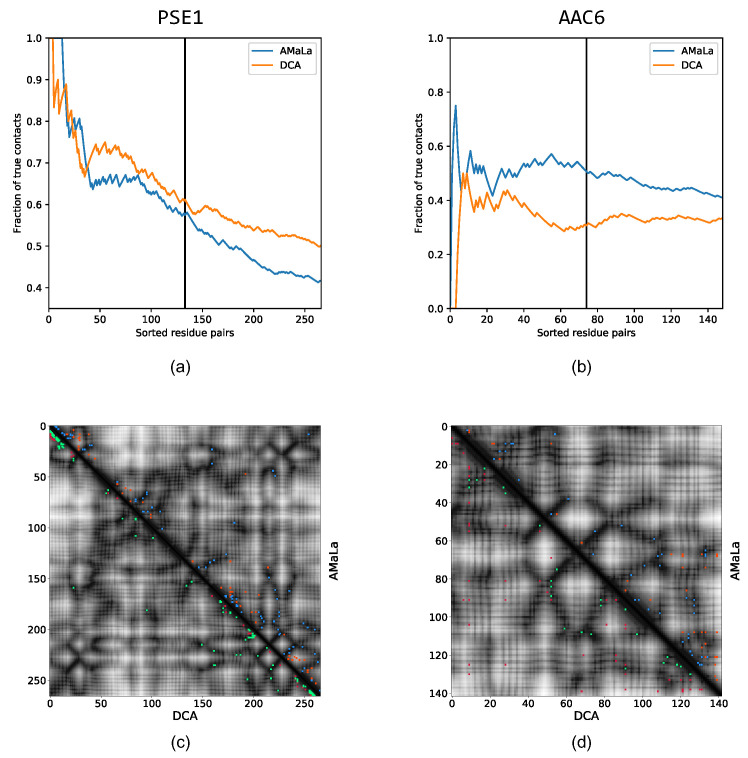
Top: sensitivity plot for contact prediction via parameters inferred on PSE-1 and AAC6 [29] datasets. Blue curve: the score is computed as the Frobenius norm of the couplings inferred with the AMaLa method. Orange curve: the score is computed as the Frobenius norm of the couplings inferred with the standard pseudo-likelihood maximization approach. In panel (**a**), we have the result for PSE-1. At the L/2-th ranked residue pair, AMaLa provides AUC(L/2)=0.71, PPV(L/2)=0.58, whereas PlmDCA yields AUC(L/2)=0.72, PPV(L/2)=0.61. Panel (**b**) shows the sensitivity plot for AAC6. In this case, AMaLa yields at half of the length AUC(L/2)=0.51, PPV(L/2)=0.51, whereas for PlmDCA, we have AUC(L/2)=0.34, PPV(L/2)=0.31. Bottom: contact maps up to L/2 predictions. In the upper-right half, the results related to AMaLa are reported, whereas in the lower-left, the prediction provided by PlmDCA is reported. Correctly predicted contacts are colored in green/blue, while wrong prediction are reported in red/orange for PlmDCA/AMaLa, respectively. Panel (**c**) reports the result for PSE-1. Even if DCA provides both higher AUC and PPV, AMaLa seems to predict more long range contacts. A similar outcome, although less pronounced, can be appreciated in panel (**d**), which shows the contact map related to AAC6.

We assessed the quality of the DCA scores derived from AMaLa and PlmDCA (the inference method used both in [26,29]) by comparing the predicted contact map with the true one obtained by the PDB structure of the protein (see Materials and Methods). The results are shown in Figure 3: From the sensitivity plots, we see that, independently from the inference strategy, the predictions for PSE-1 are more accurate than the ones for AAC6. However, if we concentrate on the AAC6 case, AMaLa predictions turn out to be more accurate. As the study of controlled artificial datasets presented seem to indicate, we expect AMaLa to provide better results with respect to PlmDCA when two conditions occur: (i) selection has a relatively weak effect compared to mutation and (ii) not too many rounds of the experiment are performed, so the Jukes–Cantor modeling of the mutation process remains a good approximation (see next section on in silico data). The first of the two conditions certainly holds for both proteins since the antibiotic concentration is slightly above the minimum inhibitory one (6 μg/mlfor PSE-1 and 10 μg/mL for AAC6), while mutation rates are approximately the same for the two. Consequently, since the PSE-1 experiment takes place over 20 rounds, while AAC6 just over 8, we expect to obtain better results in comparison with PlmDCA for the latter rather than the first. Nonetheless, the results obtained on PSE-1 show comparable precision of the two approaches, with AMaLa correctly predicting a higher number of contacts before the first error. Moreover, looking at the predicted contact map, the contacts predicted by PlmDCA are mainly close to the polypeptide backbone, while the AMaLa ones are spread over all the contact maps, providing long-range predictions that are more important for constrained molecular-dynamics simulations.

In complete analogy with what was already observed in [26], when the same approach is used for the TEM-1 dataset of Fantini et al., neither model is able to provide statistically relevant contact predictions (see Supplementary Materials Section S4 The reason can be related to the different choice of the trade-off between selection strength and mutation rate compared to Stiffler et al., as pointed out in [46]. It is remarkable that, while the model correctly predicts the fitness direct measurements, as shown in Figure 2, it fails at providing structural information.

Interestingly, in [29], the authors report that the ep-PCR introduces approximately 3–4% amino acid substitutions per round, from which we can estimate a mutation rate of $p_{\mathrm{true}}≃0.035$. We can compare it with the maximum-likelihood values inferred by AMaLa, which are $p_{\mathrm{infer}}=0.05$ for PSE-1 and $p_{\mathrm{infer}}=0.055$ for AAC6, both comparable with the experimentally estimated one.

As a further check, we decided to employ PlmDCA to infer the energy landscape not only on the last round but on all the available ones. The results in terms of contact prediction on [29] data are reported in Supplementary Materials Section S4 Table S3 From the reported values, it emerges how extending PlmDCA inference over all sequenced rounds does not provide any significant advantage but rather seems to produce a worse result.

### 3.2. In Silico Directed Evolution Experiments

The results on AAC6, PSE-1 and TEM-1 clearly indicate how different experimental conditions (in particular the choice of the mutation rate and the selective pressure) impact the ability of the inference algorithm to predict either functional and structural properties. In particular, the interplay between the mutation rate and the selective pressure determines the different dynamical regimes where the assumptions at the basis of our inference method could be more or less verified. To understand the limits of AMaLa, we simulated in silico Directed Evolution experiments at different ranges of selection and mutation rates (see details in Section 4.4). We stress here how these simulations do not aim at reproducing an actual evolution process related to a specific protein, as it is, for instance, conducted in [58,59]. Rather, the question we want to answer is: supposing that the fitness landscape is encoded into a generalized Potts-model Hamiltonian, which is known a priori, how does the inference capability of the method change with the fictitious experimental conditions (e.g., selective pressure, mutation rate, number of performed rounds)? The two main parameters of the simulated data are: (i) the site mutation probability parameter *p* and (ii) the strength of the selective pressure $\tilde{\beta }$: increasing it, the selective pressure increases.

In all experiments, we keep the teacher energy parameters and the initial *wild-type* sequence (or more precisely the ground truth energy of such sequence) fixed. We used a subset of variable sizes among the total simulated rounds (typically including between 2 and 5 rounds). The performance of the inference is assessed in terms of the correlation between teacher and student energies computed over a test set of sequences not used to train the model.

**Figure 4:**
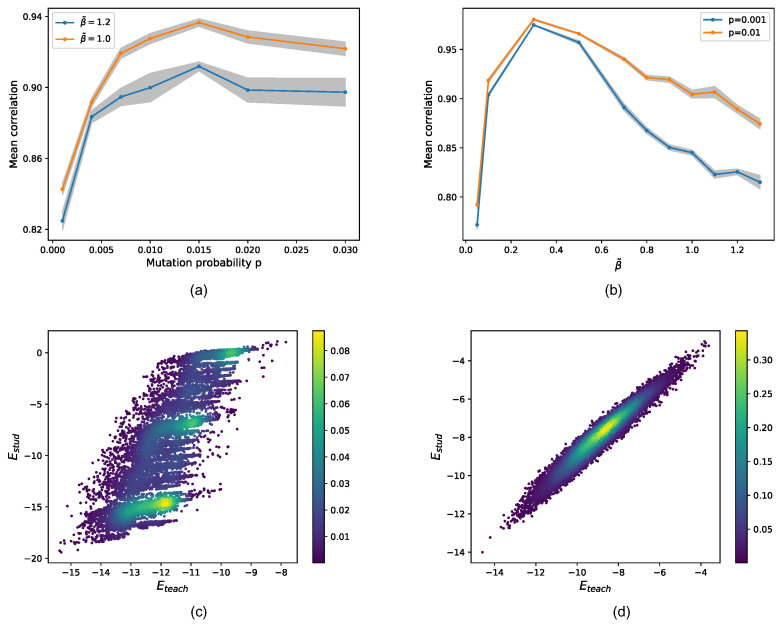
Simulated experiments varying the mutation rate and the selective pressure. In the top panels, the Pearson correlation between true and predicted fitness or equivalently teacher and student model energy are shown. In order to estimate statistical fluctuation on the correlation, for each point, several experiment replica have been realized ($N_{\mathrm{sim}}=20–40$), reporting the mean and standard deviation. In panel (**a**), the mutation rates for two choices of the selective pressure are compared: ${\tilde{\beta }}_{\mathrm{high}}=1.2$ (blue); ${\tilde{\beta }}_{\mathrm{low}}=1.0$ (orange). Conversely, in panel (**b**), the selective pressure at two fixed mutation rates is shown: $p_{\mathrm{low}}=0.001$ (blue); $p_{\mathrm{high}}=0.01$ (orange). An optimal mutation rate seems to emerge with the mean Pearson coefficient, which flattens for higher mutation rates. Again, performances appear to decrease with increasing selective pressure. Moreover, the curve coinciding with $p_{\mathrm{high}}$ displays significantly higher correlations. The bottom panels show two examples of a density scatter plot between true (*x*-axis) and inferred (*y*-axis) energies over the test set. Two limiting cases are shown: high selective pressure and low mutation rate in panel (**c**) (p=0.001 and $\tilde{\beta }=1.2$) and low selective pressure and high mutation rate in panel (**d**) (p=0.05 and $\tilde{\beta }=0.5$), where AMaLa recovers the right fitness landscape. In the former case, the Pearson’s correlation is ρ=0.81, while in the latter, it is ρ=0.97.

In Figure 4, we display the retrieval of the true fitness as a function of the mutation rate (panel (a)) and selective pressure (panel (b)). In both cases, we observe the existence of an optimal value for both tuned parameters pressure. Interestingly, above the optimal mutation rate, the correlation tends to flatten at a value that is not far from the optimal one, ensuring that AMaLa’s sweet spot for inference (at fixed selective pressure) is in general towards a high mutation rate regime. Just as a reference to real Directed Evolution experiments, the mutation rate reported in [29] is $p_{\mathrm{true}}≃0.035$.

Unfortunately, we do not have access experimentally to a quantitative assessment of the strength of the selective pressure, making a direct comparison with other experiments difficult. The method works at intermediate selective pressure as the selection tends to undermine the method assumptions (see Section 2). Indeed, when the selection strength is too low (depending on the time scale of the experiment), the sequence dynamics are dominated by genetic drift and, not surprisingly, the correlation between the teacher and student degrades. The degradation of the performance observed for higher selective pressure is due to a combination of effects: on the one hand, we expect the in the limit of β→∞, only the lowest energy sequence generated in the mutation step would survive, making any inference impossible. On the other hand, at intermediate but high selective pressure, we expect that the consensus sequence starts drifting significantly from the initial wild-type sequence, making the drift term of Equation (7) an inaccurate description of the purely mutational step.

In Directed Evolution experiments, one of the limiting factors is the number of selection rounds that can be sequenced (and that therefore can be used for the inference). In the following, we will assume we can afford only between two and five rounds of sequencing, and we ask which rounds bring the larger information content.

**Figure 5:**
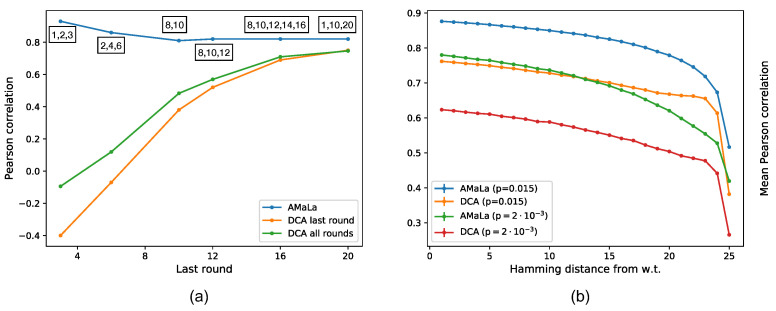
Inferred signal dependence on the number of rounds and hamming distance from wild-type. Left panel (**a**): Pearson correlation when the number of rounds are changing. Comparison between PlmDCA on the last round (or all rounds) and AMaLa. The sequenced rounds are: (1, 2, 3); (2, 4, 6); (8, 10); (8, 10, 12); (8, 10, 12, 14, 16); (1, 10, 20). PlmDCA significantly depends on the number of performed rounds, not significantly inferring the fitness landscape up to round ∼16. On the contrary, AMaLa provides predicted energy functions highly correlated with the fitness even for a low number of performed selection rounds. Right panel (**b**): Degradation of the mean Pearson correlation between inferred and true energies as a function of the Hamming distance from the wild-type sequence. Two different simulations are considered: high (p=0.015) and low (p=0.002) mutation probability. Changing such parameters varies the broadness of the library screening during an experiment, resulting in probing a more local or more broad region of the sequence space. AMaLa predictions are systematically better than PlmDCA, while the latter displays a slower decrease in correlation augmenting the distance from wild-type.

As shown in Figure 5 panel (a), for PlmDCA, the correlation between the teacher and student energies of the test set increases as a function of the last round time, whereas AMaLa performance behaves just in the opposite way: earlier round times give better results. This finding is particularly interesting as it suggests that by using AMaLa, one could achieve better inference results by performing just a limited number of rounds, i.e., with lower experimental effort. However, AMaLa’s overall performance is always better than PlmDCA for any sequencing round.

Furthermore, the in silico experiments can be used to investigate the generalization power of the learned fitness landscape beyond the local region of sequence space probed by the experiment. More specifically, how far from the wild-type an inference strategy is still able to predict the fitness? To answer this question, we trained both AMaLa and PlmDCA on rounds (2,4,8). Then, we tested the teacher–student energy correlation over randomly extracted sequences at a Hamming distance up to the whole sequence length (here L=25). As shown in Figure 5 panel (b), we can see that that both in the case of low and high mutation rates: (i) over the whole range of Hamming distances from the wild-type sequence, AMaLa always shows a higher correlation with the teacher energies; (ii) PlmDCA’s performance seems to degrade more slowly as a function of the distance from the wild-type sequence.

## 4. Materials and Methods

### 4.1. Experimental Pipeline

**Figure 6:**
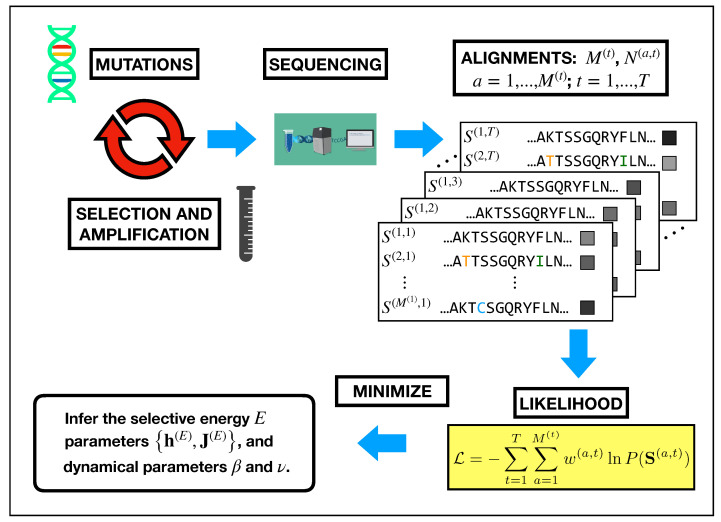
Pictorial representation of data generation in a Directed Evolution experiment and how they are plugged into the likelihood function to perform the inference. The sequencing of repeated rounds of mutation and selection generates a set of multiple sequence alignments. There, we highlighted with colored letters the sites that have been mutated with respect to the wild-type, coinciding with the first row of the alignment. Moreover, at the right of the sequences, boxes in gray-scale represents the abundances (increasing from black to white). Each sample of sequences $\left\{S^{\left(t\right)}\right\}$ and the related abundances $\left\{N^{\left(t\right)}\right\}$ for t=1,⋯,T are used to define the likelihood function, which subsequently depends only on the parameters to be inferred: $\theta_{H}=\left\{h^{\left(E\right)},J^{\left(E\right)},\nu ,\beta \right\}$ (see Equations (1)–(3) and (7)). The inference of these parameters is based on the maximization of the log-likelihood. In order to determine the parameters β and ν, the maximization problem over the energetic parameters is repeated, performing a scan over a set of possible values. Then the pair $\left(\beta_{\mathrm{opt}},\nu_{\mathrm{opt}}\right)$ corresponding to the global maximum of the minus log-likelihood is retained.

The experiments involve repeated rounds of mutation and selection, starting from a natural sequence, named *wild-type*. Repeated cycles of error-prone PCR are applied to the library at each round to introduce mutations. Functional selection is obtained by inserting the plasmids with the variants in a bacterial colony and then placing the colony in an environment with a relatively low antibiotic concentration: 6 μg/mL ampicillin for PSE-1 and 10 μg/mL kanamycin for AAC6. Conversely, two different ampicillin concentrations are used for TEM-1, namely 25 μg/mL for all rounds but 5 and 12, when the concentration is raised to 100 μg/mL. For a subset of the rounds (with the last round included), a sample of the population after the selection is sequenced. Thus, for each sequenced round *t*, we obtain the abundances $N^{\left(a,t\right)}$ for the variants $a=1\cdots ,M^{\left(t\right)}$, with a.a. sequence $S^{\left(a,t\right)}$, $M^{\left(t\right)}$ being the number of unique sequences present in the sample at time *t* (see the Figure 6 for the full pipeline).

Raw data related to experiment [26] are available in the National Centre for Biotechnology Information Sequence Read Archive (SRA), therein accessible via the code PRJNA528665 (http://www.ncbi.nlm.nih.gov/sra/PRJNA528665). More refined data can be found at BioSNS site: http://laborator- iobiologia.sns.it/supplementary-mbe-2019/. Raw data related to [29] can also be found in SRA, with accession code PRJNA578762, whereas refined data can be downloaded from https://github.com/sanderlab/3Dseq. Both datasets were downloaded on 12 June 2020.

### 4.2. Model Learning

The abundances of the sequenced variants are used to compute the normalized weights $w^{\left(a,t\right)}$ in Equation (2). To learn the parameters $\left(\nu ,\beta ,\theta_{E}\right)$ of the Model (7), we maximize Equation (2) using a pseudo-likelihood approximation (see Supplementary Materials Section S1 Although Equation (2) is convex with respect to the energy parameters $\theta_{E}$ and the inverse temperatures β separately, it is no longer true when the parameters are varied simultaneously. Two strategies are possible: (i) optimizing the energetic parameters at different values of β and select the maximal pseudo-likelihood. (ii) Starting from an arbitrary β and sequentially optimizing $\theta_{E}$ and β using the gradient descent algorithm (e.g., Newton algorithm). Since the $\theta_{E}$ is defined up to a multiplicative constant, we can set $\beta (t_{1})=1$ (with $t_{1}$ the time of the first sequenced round) or alternative β(t=T)=1 without losing generality. The last choice is mandatory in the case in which β(T) diverges. In such a scenario, $\beta (t_{1})\equiv 0$, and the intermediate values are constrained in the domain [0,1]. In order to set an optimal value for the Jukes–Cantor parameter ν(t), we decided to maintain the functional form in Equation (6), thus performing a scan over the possible values of the mutation rate μ. The value $\bar{\mu }$ yielding a maximum for the pseudo-likelihood then defines each component for the different round time: $\nu \left(\bar{\mu }\right)=\left(\nu \left(\bar{\mu },t_{1}\right),\cdots ,\nu \left(\bar{\mu },T\right)\right)$.

### 4.3. Contacts Prediction

To compute an epistatic score associated with each pair of residues, we used the Frobenius norm with APC correction (originally introduced in [60]. For each pair of positions *i* and *j*, the Frobenius norm over all possible amino acid combinations of the interaction parameters $J_{ij}$ is computed:

(8) $$F_{ij}=\sqrt{\sum_{a,b=1}^{q-1}J_{ij}{\left(a,b\right)}^{2}}\;.$$

As the Frobenius norm is not-gauge invariant, it is important to transform the Potts parameters in zero-sum-gauge first [57]. Lastly, we applied the average product correction (APC) to the *F* matrix: $F_{ij}^{APC}=F_{i,j}-F_{i,\cdot }F_{\cdot ,j}/F_{\cdot ,\cdot }$, where the dot represents the average over the index. The same procedure has been used to obtain DCA scores derived from both AMaLa and PlmDCA.

To assess the predicted contact maps, we compared it to the residues contact extracted from crystal structures present in the PDB database (1G68 for PSE-1, 4EVY for AAC6 and 1ZG4 for TEM-1). Two residues are in contact if at least two heavy atoms have a distance less than 8Å. We only consider residues with a separation on sequence |i−j|≥5.

### 4.4. Experiment Simulation

To simulate a Directed Evolution experiment, we define a dynamical process that mimics the mutation and selection steps occurring in a real experiment. We define $N^{\left(a,t\right)}$ as the number of clones of variant *a* present at the round *t* for t∈{1,⋯,T}. The total number of clones is kept fixed along the simulation and equal to $\sum_{a=1}^{M^{\left(t\right)}}N^{\left(a,t\right)}=N_{\mathrm{tot}}=2\cdot 10^{7}$.

Mutations are drawn from a site-independent uniform distribution over the space of 20 amino acids. The unique parameters we consider is the mutation probability *p* (or equivalently the mutation rate μ see Supplementary Materials Section S2 For every clone of a given variant, the number of sites to be mutated is drawn from a binomial distribution of probability *p*.

(9) P(#mut=k)=Lkpk(1−p)L−k.

In practice, for each selected site, the new mutations are uniformly extracted over the possible different amino acids. This process either generates new variants or increases the abundances of already present ones.

Finally, we simulate the selection step by associating a *survival* probability $P_{S}\left(S^{\left(a\right)}\right)$ to each variant *a* via a Boltzmann weight proportional to $\exp-\tilde{\beta }\left[E_{\mathrm{teacher}}\left(S^{\left(a\right)}\right)-\tilde{\mu }\right]$.

The energy function $E_{\mathrm{teacher}}$ has the same functional form of Equation (3) and given its parameters, it constitutes the ground-truth fitness landscape (the *teacher* model). The parameter $\tilde{\mu }$ is a sequence-independent chemical potential that fixes the scale of the binding probability. Typically, numerical values employed in the simulations are around $\tilde{\mu }∼-18.6$.

From the set of variants produced by the mutation process, which we identify as ${\left\{{\tilde{N}}^{\left(a,t\right)}\right\}}_{a=1}^{{\tilde{M}}^{\left(t\right)}}$ (${\tilde{M}}^{\left(t\right)}$ is the number of unique sequences after the mutation step only), a subset $n^{\left(a,t\right)}$ of surviving clones is selected according to a binomial process defined by:

(10) PB(n(a,t)|N˜(a,t))=N˜(a,t)n(a,t)PS(S(a))n(a,t)(1−PS(S(a)))N˜(a,t)−n(a,t).

Finally, the population of clones that survived the selection step is amplified up to a fixed number $N_{\mathrm{tot}}$ according to the following multinomial distribution:

(11) $$P_{A}\left(N^{\left(t\right)}|n^{\left(t\right)}\right)=\frac{N_{\mathrm{tot}}!}{\prod_{a^{\prime }=1}^{M^{\left(t\right)}}N^{\left(a^{\prime },t\right)}!}\prod_{a=1}^{M^{\left(t\right)}}{\left(\frac{n^{\left(a,t\right)}}{n_{\mathrm{tot}}}\right)}^{N^{\left(a,t\right)}}.$$

In addition, we randomly sample $R_{\mathrm{tot}}=10^{6}$ sequences out of the $N^{\left(t\right)}$ present variants to introduce the sampling noise and simulate the effect of the sequencing.

In Figure S1 in the Supplementary Materials, a pictorial representation of the whole pipeline for the generation of simulated data is reported.

The parameters setup of the simulation was chosen with the aim to be as close as possible to a real experiment and to not introduce unnecessary artificial features. The teacher model for the ground truth fitness landscape is obtained by the inference of a Potts model on a Deep Mutational Scan (DMS) experiment [61]. The inference method used to obtain the teacher model is described in [53], and it provides a reliable model of the fitness in the absence of mutagenesis steps. In the considered (DMS) experiment, the WW domain of the hYAP65 protein has been mutated and selected to bind to its cognate peptide ligand. The mutated part of the protein has a length L=25 amino acids.

While finalizing this work, we became aware of a similar approach described in [46]. Their strategy relies on a simultaneous treatment of selection and mutagenesis. The fitness approximated landscape is inferred over the homologous alignment, specifically via Boltzmann learning of a generalized Potts model. Such energy provides a proxy for fitness and a tool to probe context-dependent mutations, as the energy function includes couplings between different residues. Indeed, an MCMC is implemented to generate a library that mimics the one that would have been obtained in a real Directed Evolution experiment. The elementary step of this MCMC includes both mutation and selection. The energy variation of single-site mutations with respect to the wild-type defines the acceptance probability (which depends only on the a.a. sequence). On the other hand, the proposed mutations are restricted to the allowed single mismatch transitions among codons $c^{i}=\left(c_{1}^{i},c_{2}^{i},c_{3}^{i}\right)$, thus involving the genomic sequence. This may suggest a possibility to improve AMaLa itself: the Hamming distance in the Jukes–Cantor contribution in Equation (7) may be computed over the genome alignment. In this way, forbidden transitions among a.a.’s are automatically excluded, but at the same time, multiple transitions are also allowed, even if exponentially suppressed. Remarkably, the findings of [46] with respect to the optimal regime for a Directed Evolution experiment agrees with the results we derived from the application of AMaLa to both in silico and in vitro data.

## 5. Conclusions

In this work, we presented AMaLa, a new inference strategy for modeling Directed Evolution experiments. At the heart of our algorithm, there is an effective model of the two main ingredients of Directed Evolution experiments: mutation and selection. Other competing algorithms’ computational strategies typically use data from the last sequenced round, whereas our model leverages all the available *history* of the experiment in terms of all sequenced rounds of selection. By doing so, we are able to infer a better statistical model both in terms of the ability to predict functional phenotypes and structural properties of the protein.

As Directed Evolution is becoming a very relevant instrument to test different evolutionary theories on a controlled ground, as well as an invaluable tool to find optimal target phenotype sequences with pharmaceutical and/or biotechnological interest, we believe that a reliable statistical modeling of the experiment has two-fold interest: on the one hand, we show how our model can quantitatively predict the trait under selection of variants that have not been used in the training data, suggesting that our model could, in principle, be used to propose sequences and/or libraries of sequences of improved biological activity. On the other hand, our statistical model could be used to optimally set the experimental control parameters. In particular, we were able to stress how relevant the trade-off between mutation and selection is in different experiments. Our findings are also corroborated by extensive in silico Directed Evolution experiments, where the modification of these two parameters can easily be taken into consideration in limits that would otherwise be experimentally inaccessible.

AMaLa, of course, is a first attempt at modeling evolutionary trajectories under fixed selection pressure, although some interesting attempts have been recently published in [62] in the somehow different context of the inference of genetic linkage in population genetics. In particular, the way in which we model the mutation step could be made more accurate by taking into account codon biases and more realistic transition probabilities (the first attempt in this direction has been proposed in [46]). From this point of view, our work suggests that it might be useful to sequence the library before and after the selection step to disentangle the effect of mutation and selection and produce a better correlation between statistical energies and the empirically measured trait under selection.

## Data Availability

Publicly available datasets were analyzed in this study. This data can be found here: https://www.ncbi.nlm.nih.gov/sra/PRJNA528665, http://laboratoriobiologia.sns.it/supplementary-mbe-2019, https://www.ncbi.nlm.nih.gov/sra/?term=PRJNA578762, https://github.com/sanderlab/3Dseq, https://gitlab.com/luca.sesta/Amala.jl, https://doi.org/10.1093/molbev/msu081, https://www.pnas.org/content/suppl/2013/07/18/1215206110.DCSupplemental.

## Supplementary Materials

Supplementary text available at https://www.mdpi.com/article/10.3390/ijms222010908/s1.

## Abbreviations

The following abbreviations are used in this manuscript:

AMaLa	Annealed mutational approximated Landscape
DCA	Direct coupling analysis
DMS	Deep mutational scanning
PCR	Polymerase chain reaction
AUC	Area under the curve
PPV	Positive predicted value
